# Robustness and capabilities of ultrashort laser pulses characterization with amplitude swing

**DOI:** 10.1038/s41598-020-75220-4

**Published:** 2020-10-27

**Authors:** Íñigo J. Sola, Benjamín Alonso

**Affiliations:** Grupo de Investigación en Aplicaciones del Láser y Fotónica, Departamento de Física Aplicada, University of Salamanca, 37008 Salamanca, Spain

**Keywords:** Ultrafast lasers, Optical techniques, Optical metrology

## Abstract

In this work we firstly study the influence of different parameters in the temporal characterization of ultrashort laser pulses with the recently developed amplitude swing technique. In this technique, the relative amplitude of two delayed replicas is varied while measuring their second-harmonic spectra. Here we study the retrieval of noisy traces and the implications of having different delays or phase retardations (relative phases) between the two replicas. Then, we study the capability of the technique to characterize the pulses when the second-harmonic signal is spectrally uncalibrated or incomplete, presenting the analytical calculation of the marginal, which is used to calibrate the traces and to perform the pulse retrievals. We experimentally show the retrieval of different pulses using diverse delays and phase retardations to perform the amplitude swing trace and demonstrate that, from an uncalibrated trace, both the pulse information and the response of the nonlinear process can be simultaneously retrieved. In sum, the amplitude swing technique is shown to be very robust against experimental constraints and limitations, showing a high degree of soundness.

## Introduction

Ultrashort laser pulses are a very demanded light source involved in many applications, for example in microscopy^1^, material processing^2^, particle acceleration^3^, nanoparticle production^4^, nonlinear processes^5,6^ or attosecond pulse generation^7^. The characterization of those pulses is a key point for the understanding and optimization of those processes.

Many different techniques have been proposed and employed for that purpose, based either on referenced or self-referenced schemes^8^. In the last decade, more compact schemes have been introduced, as in MIIPS^9^, d-scan^10^ or chirp-scan^11^. Recently, we have proposed and demonstrated the amplitude swing concept to measure ultrashort laser pulses^12^. In this technique, two delayed replicas of the input pulse are created with different relative amplitudes, while the spectrum of their second harmonic (SH) generation is measured for the different amplitudes, thus resulting in a two-dimensional trace. We showed how a rotating multiple-order waveplate (MWP) –a birefringent plate with appropriate thickness– can be used to implement the technique in a very simple and compact layout.

In this work, we theoretically study the impact on the pulse measurement of two key parameters in amplitude swing: the temporal delay between the replicas and the phase retardation between the MWP axes. Both parameters are important as they are indicating the validity of a particular system to be used with pulses of different bandwidth (Fourier-limit) and spectral range. We also study the influence of the presence of noise in the SH trace.

Regarding the temporal duration of the pulses, few- and single-cycle light sources^13,14^ have many applications, e.g. in attosecond pulses, acceleration or atomic physics. The characterization of these pulses involves measuring over large bandwidths. This may require a trade-off in the phase-matching bandwidth. In other techniques, this point has been addressed with different strategies, for example in FROG with spectral correction^15^. The SPIDER technique has also been shown to operate in the few-cycle regime^16^. In the d-scan technique, the calibration of the trace can be directly done with the frequency marginal^10^. Correspondingly, self-referenced techniques measuring two-dimensional traces may present redundancy in the data. In this way, ptychographic algorithms have been used to reconstruct incomplete FROG traces^17^, and the d-scan has also been shown to retrieve the pulse with incomplete traces^18^.

In the present work, we study the capability of amplitude swing to retrieve incomplete SH traces, with spectral clipping or presenting hollow regions. Redundancy in the data arises from measuring a two-dimensional trace to retrieve a one-dimensional pulse and, what is more, from the fact that SH is effectively a sum of frequencies for the whole bandwidth of the input pulse. Therefore, a certain nonlinear frequency has information of many fundamental frequencies. Thanks to this, we find that the pulse can be correctly retrieved for clipped traces to some extent.

Concerning the measurement of pulses with uncalibrated traces –due to non-flat SH amplitude response–, we propose to use the frequency marginal of the amplitude swing trace to calibrate itself. We find that this marginal depends on the spectral phase of the pulse to be retrieved, which prevents its direct calculation. In the Appendix, we demonstrate the analytical expression of the marginal, which we use to theoretically and experimentally demonstrate that it can be used to calibrate the trace during the pulse retrieval algorithm.

In the next section, we describe the basis of the amplitude swing technique and introduce the idea of calibrating the trace with the frequency marginal. Then, we present the results for the numerical simulations to study different parameters and dependences and we theoretically demonstrate the retrieval of incomplete or uncalibrated traces. Finally, we experimentally demonstrate the pulse retrieval for differently chirped pulses, using different delays and phase retardations, and we show the pulse retrieval under a non-flat SH response.

### Amplitude swing technique and retrieval for incomplete traces

**Figure 1:**
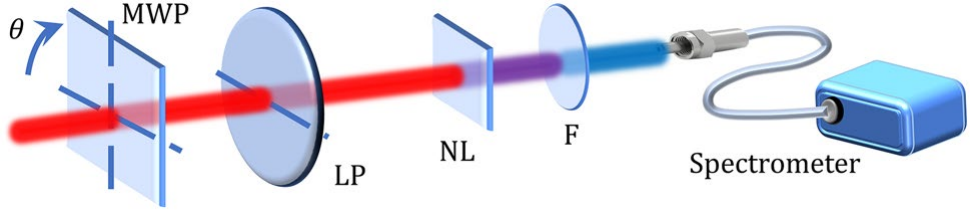
Scheme of the experimental setup for the amplitude swing technique. Input horizontal linear polarization pulses impinge a multi-order waveplate (MWP). Different orientations of the MWP, followed by the selection of the horizontal component projection with a linear polarizer (LP), produces two delayed replicas of the input pulse with different relative amplitudes. Those replicas generate second harmonic in a nonlinear (NL) crystal, whose spectrum is measured after a filter (F) that eliminates the remaining linear signal.

In the amplitude swing technique^12^, two delayed replicas of the input pulse are created, then their relative amplitude is varied, and they experiment a nonlinear process (e.g., SH generation), being the amplitude of the nonlinear signal resolved in frequency with a spectrometer. A rotating birefringent material combined with a linear polarizer is used to perform the shaping of the replicas in the pulse manipulation described before. The angle of the fast axis of the MWP with respect to the *x*-axis is $\theta$. We consider the input pulse to be characterized, $E_{0} (\omega ) = A(\omega )e^{{i\phi_{p} (\omega )}}$, where $A(\omega )$ and $\phi_{p} (\omega )$ are its spectral amplitude and phase, respectively. The birefringent material can be an MWP, responsible of introducing a delay $\tau$ between the fast and slow axes, which introduce phases $\phi_{f} (\omega )$ and $\phi_{s} (\omega )$, respectively. We choose the input pulse to be linearly polarized (0°) and the fast axis of the MWP to rotate between 0 and 180°, after which the resulting pulse is projected to the *x*-axis with a fixed linear polarizer (LP) at 0°, before generating SH in a nonlinear (NL) material (see Fig. 1).

The expression for the resulting two-dimensional nonlinear spectrum signal –the amplitude swing trace– is given by the following equation

1 $$S_{SH} \left( {\omega ,\theta } \right) = \left| {\int {\left( {\int {A(\omega^{\prime } )e^{{i\phi_{p} (\omega^{\prime } )}} \left[ {e^{{i\phi_{f} (\omega^{\prime } )}} \cos^{2} \theta + e^{{i\phi_{s} (\omega^{\prime } )}} \sin^{2} \theta } \right]e^{{i\omega^{\prime } t}} d\omega^{\prime } } } \right)^{2} e^{ - i\omega t} dt} } \right|^{2}$$

The cosine and sine terms are responsible of the relative amplitude swinging of the two replicas before generating the nonlinear signal. The trace given in Eq. (1) encodes the spectral phase $\phi_{p} (\omega )$ of the pulse, which can be retrieved with a proper algorithm^12^. Here, we also use iterative optimization by means of the Levenberg–Marquardt algorithm. In the experiments, we characterize with precision the phase retardation of the MWP for the central wavelength by using inline interferometry^19^, while the global phase due to the dispersion of the fast and slow axes is calculated from the material Sellmeier equations^20^.

As said before, we performed the rotation $\theta$ of the MWP between 0° and 180°. As follows from Eq. (1), the amplitude swing trace is ideally symmetrical with respect to $\theta = 90{^\circ }$, due to $S_{{SH}} \left( {\omega ,\theta } \right) = S_{{SH}} \left( {\omega ,180{^\circ } - \theta } \right)$ within that interval. Since the MWP may not operate perfectly symmetrical in the experiments, we decided to rotate $\theta$ between 0° and 180° in order to have more robust results and to check said symmetry of the trace. In fact, the amplitude swing trace would be repeated by rotating from 180° to 360°.

In the retrieval algorithm, an arbitrary initial spectral phase (guess) is used to calculate a simulated amplitude swing trace. From the comparison between the experimental and the simulated SH traces it is defined a merit function, which is minimized to retrieve the spectral phase of the pulse. Previously^12^, the amplitude swing was used to retrieve pulses with a flat and complete spectral response of the SH.

In this work, we will also study the effect of having spectral clipping or hollow regions in the SH trace. Here, we will define the merit function only in the spectral ranges where the SH trace information is complete, showing that it is still possible to retrieve the pulse characterization. This is possible due to having a two-dimensional trace –for a one-dimensional measurement– with redundant information and because the SH is actually the sum of frequencies along the spectrum of the pulse, mixing different parts of the fundamental spectrum in several parts of the SH spectra^18^.

In the case of having an uncalibrated SH signal spectrum, we need to have a procedure to enable the retrieval of the pulse. In other techniques^10^, the frequency marginal $M_{\omega } (\omega )$ of the trace can be used to calibrate the SH response in amplitude, $R_{SH} \left( \omega \right)$. $M_{\omega }$ is defined as the integral of the amplitude swing trace over the rotation angle $\theta$ of the MWP. In the Appendix, we demonstrate the analytical expression of $M_{\omega }$ [final expression in Eq. (8)]. We find that in the case of amplitude swing, the marginal depends on the spectral amplitude of the pulse, the spectral phase of the pulse, and the dispersion of the fast and slow axes of the MWP. Thus, $M_{\omega }$ varies with the spectral phase of the pulse, $\phi_{p} (\omega )$, which is the function optimized during the retrieval. The solution that we propose and demonstrate here is to calculate and update the calibration of the SH response at every step within the iteration (a similar concept has been used before in self-calibrating d-scan to update the calibration of the dispersion inside the optimization^21^). Here, from the analytical expression of $M_{\omega }$ compared to the experimental marginal of the trace, we calculate the calibration of the SH response as

2 $$R_{SH} \left( \omega \right) = \frac{{M_{\omega ,experimental} (\omega )}}{{M_{\omega ,analytical} (\omega ,\left. {\phi_{p} (\omega )} \right|_{current} )}} = \frac{{\int_{\theta } {S_{SH,experimental} \left( {\omega ,\theta } \right)} d\theta }}{{\int_{\theta } {S_{SH,analytical} \left( {\omega ,\theta } \right)} d\theta }}$$

We will theoretically demonstrate through numerical simulations that the procedure here exposed is suitable for the simultaneous retrieval of the pulse and calibration of the response $R_{SH} \left( \omega \right)$. Additionally, we will also present the experimental demonstration when using a thicker nonlinear crystal having non-flat spectral response along the spectral bandwidth of the pulse (results in Fig. 8).

## Results and discussion

We performed different sets of systematic simulations to study their possible influence to encode and/or to retrieve the pulse information. We considered a simulated pulse with gaussian spectrum (central wavelength of 800 nm, bandwidth of 18.8 nm full-width at half maximum, FWHM) corresponding to a Fourier limited temporal intensity of $\tau_{\small FL}$ = 50 fs (FWHM). The simulated spectral phase of the pulse, $\phi_{p} (\omega )$, consisted in a combination of second- and third-order dispersion of—8000 fs^2^ and + 20,000 fs^3^, respectively, thus the pulse duration corresponded to $\tau_{p}$ = 179.8 fs (FWHM). The choice of these dispersion values is motivated by the previously reported fact that for very high chirp values may more subtle encoded in the amplitude swing trace^12^, so that we decided to perform the study under adverse conditions.

Below, we present the effect of noise, different phase retardations or time delays between the pulse replicas, spectral clipping or hollow regions, and, finally, uncalibrated traces. The MWP is simulated to be a quartz plate with a phase retardation for the central wavelength $\phi_{0,fs} = \pi + 2m\pi$ (half-wave plate operation), and with 1.5-mm thickness introducing a delay $\tau$ = 50 fs ($\tau = \tau_{\small FL}$) between the fast and slow axes. Hereinafter, we will omit the implicit $+ 2m\pi$ that is always present in the phase retardation of the MWP. Notice that the phase retardation and the delay (thickness) will be different when analysing their respective effects.

### Robustness against noise

Here, we consider the effect of having a noisy amplitude swing trace by adding white Gaussian noise to the simulated trace. We have added noise in a range from 0 to 8% (defined as the root-mean-square, RMS, of the noise to signal) with respect to the normalized SH trace.

**Figure 2:**
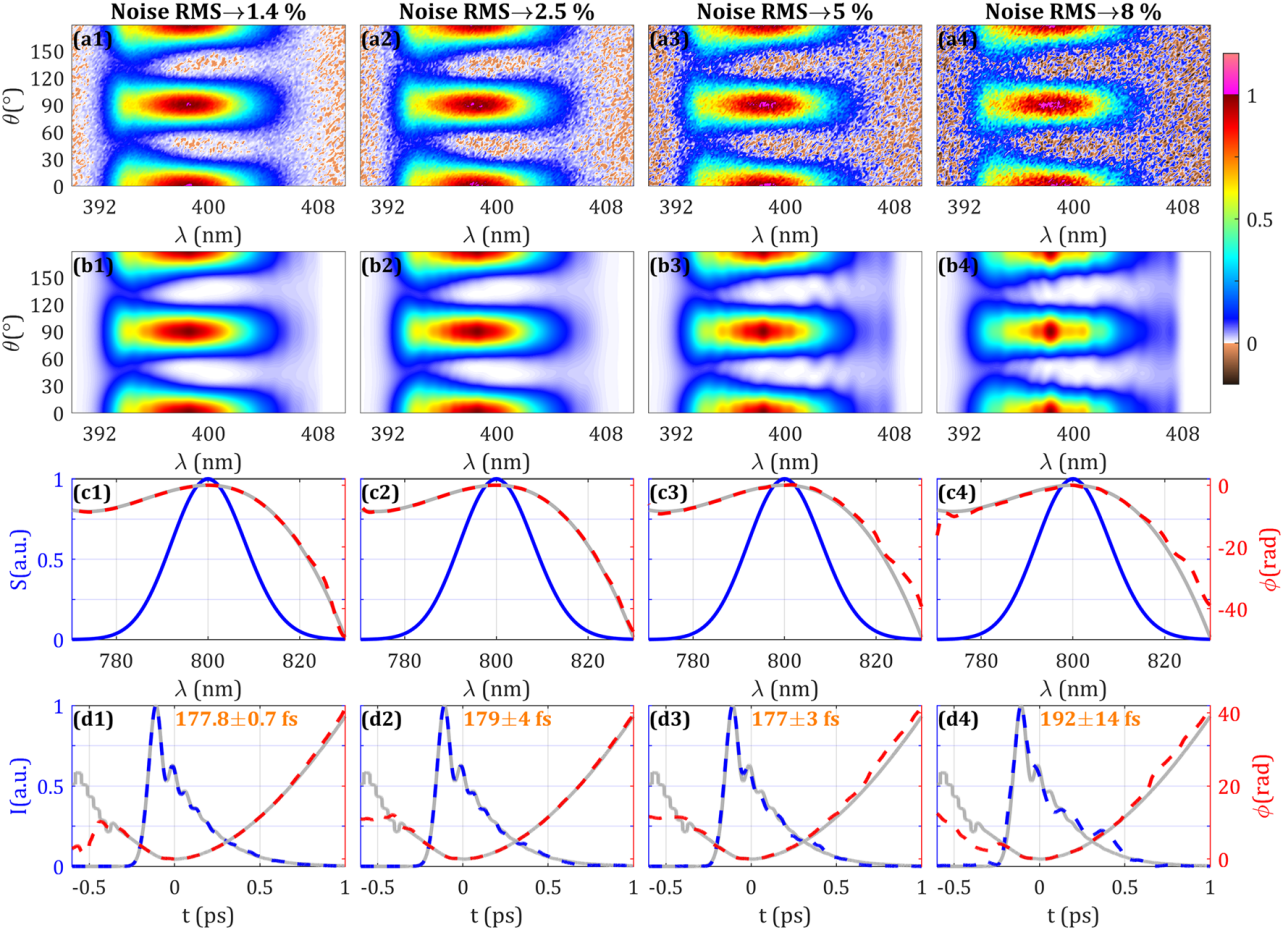
Effect on the SH trace of different noise levels is represented in Columns 1–4. (Row **a**) Simulated and (Row **b**) retrieved amplitude swing traces. (Row **c**) Spectrum (blue), simulated (grey) and retrieved (dashed red) spectral phases. (Row **d**) Simulated temporal intensity and phase (grey), retrieved temporal intensity (dashed blue) and phase (dashed red). Inset: retrieved intensity FWHM. Evolution and expanded results with other noise levels can be seen in Supplementary Video 1.

**Figure 3:**
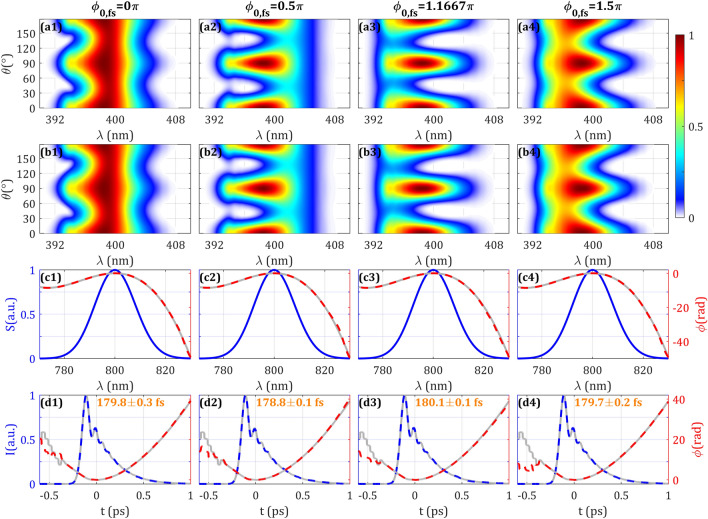
Effect on the SH trace of different phase retardations for the central wavelength of the MWP is represented in Columns 1–4. (Row **a**) Simulated and (Row **b**) retrieved amplitude swing traces. (Row **c**) Spectrum (blue), simulated (grey) and retrieved (dashed red) spectral phases. (Row **d**) Simulated temporal intensity and phase (grey), retrieved temporal intensity (dashed blue) and phase (dashed red). Inset: retrieved intensity FWHM. Evolution and expanded results with other phase retardations can be seen in Supplementary Video 2.

**Figure 4:**
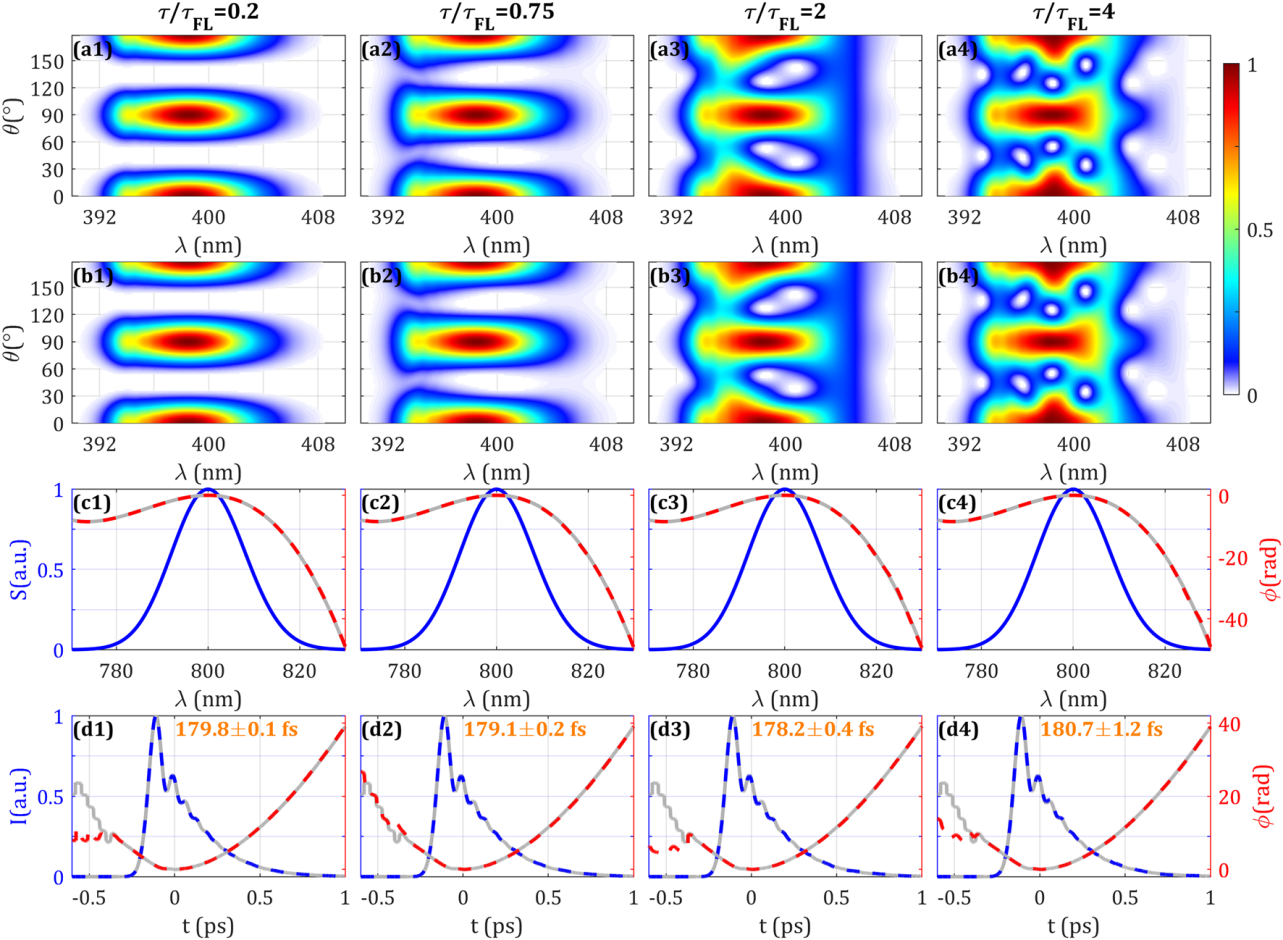
Effect on the SH trace of different temporal delays (due to different plate thicknesses) introduced by the MWP is represented in Columns 1–4. (Row **a**) Simulated and (Row **b**) retrieved amplitude swing traces. (Row **c**) Spectrum (blue), simulated (grey) and retrieved (dashed red) spectral phases. (Row **d**) Simulated temporal intensity and phase (grey), retrieved temporal intensity (dashed blue) and phase (dashed red). Inset: retrieved intensity FWHM. Evolution and expanded results with other delays can be seen in Supplementary Video 3.

**Figure 5:**
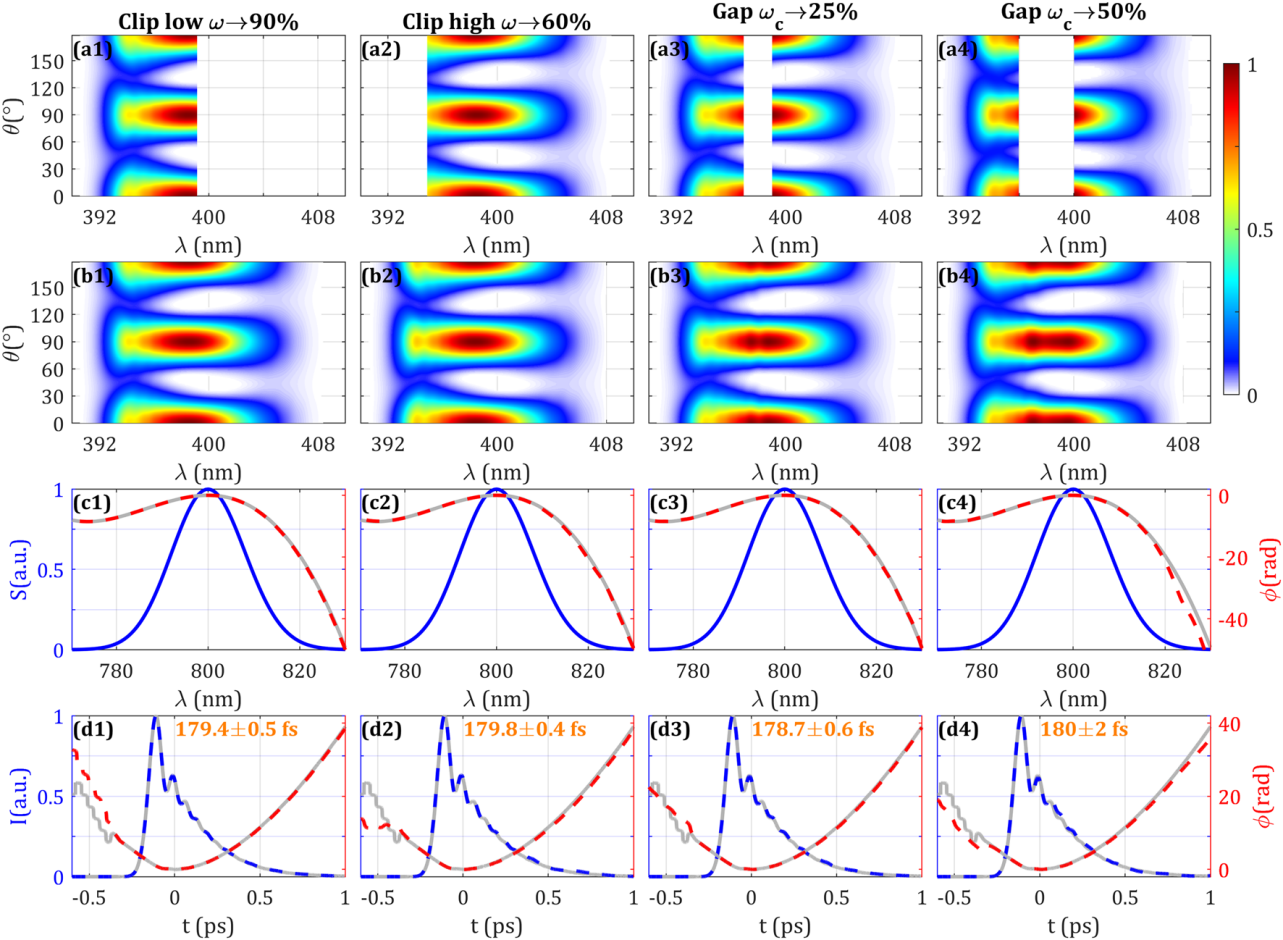
Effect on the SH trace of spectral clipping and hollow regions: low (Column 1) and high (Column 2) frequency clipping, and hollow central regions (Columns 3 and 4). (Row **a**) Simulated and (Row **b**) retrieved amplitude swing traces. (Row **c**) Spectrum (blue), simulated (grey) and retrieved (dashed red) spectral phases. (Row **d**) Simulated temporal intensity and phase (grey), retrieved temporal intensity (dashed blue) and phase (dashed red). Inset: retrieved intensity FWHM. Evolution and expanded results can be seen in Supplementary Videos 4, 5 and 6, for low frequency clipping, high frequency clipping, and hollow central regions, respectively.

**Figure 6:**
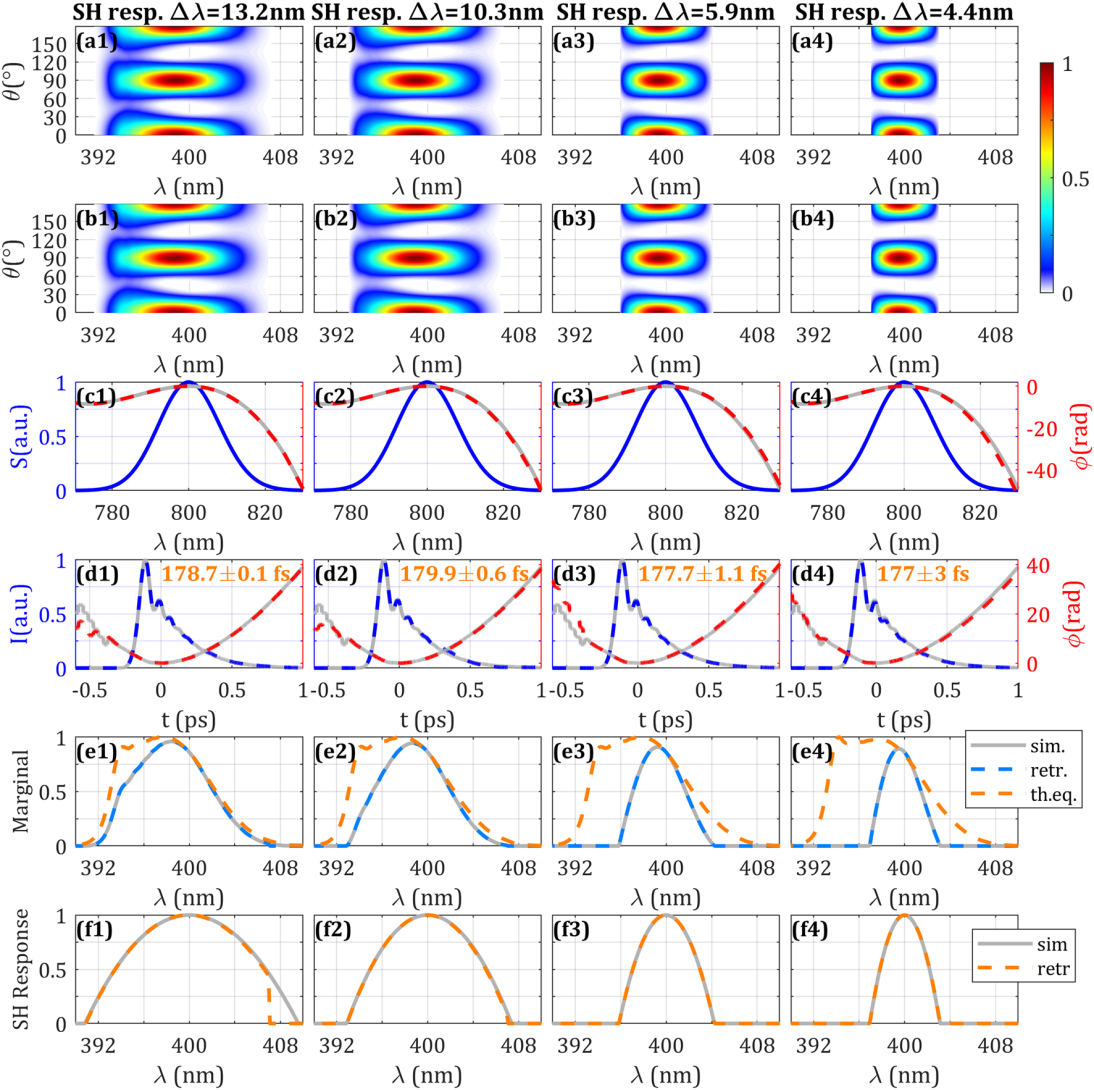
Effect on the SH trace of uncalibrated amplitude due to non-flat SH response is represented in Columns 1–4. (Row **a**) Simulated and (Row **b**) retrieved amplitude swing traces. (Row **c**) Spectrum (blue), simulated (grey) and retrieved (dashed red) spectral phases. (Row **d**) Simulated temporal intensity and phase (grey), retrieved temporal intensity (dashed blue) and phase (dashed red). (Row e) Marginal of the simulated measured trace shown in row a (grey) and marginal of the retrieved trace shown in row b (dashed blue) integrating over the rotation angle, compared to the retrieved marginal using the analytical expression and the retrieved spectral phase (dashed orange). (Row f) SH response applied to the simulated trace (grey) and retrieved SH response using the marginals (dashed orange). Inset: retrieved intensity FWHM. Evolution and expanded results with different levels of SH response can be seen in Supplementary Video 7.

We show in Fig. 2 the results for a selection of different noise levels (more values shown in Supplementary Video 1), including the simulated trace, the retrieved trace, the retrieved spectral phase, and the retrieved temporal intensity and phase. In the results presented in Figs. 2–6, we performed 5 retrievals for each trace using different random second and third order dispersion values for the guess spectral phase. The retrieved pulse intensity duration (Fig. 2, row d) is the average of the 5 retrievals and the statistical error is given by the standard deviation. As the noise level increases, it is seen that the quality of the convergence of the retrieved trace is worst, finding pulse durations errors < 0.5% for noise RMS < 1.5%, errors ≈2% for noise RMS ≤ 5 and the statistical error increases to ≈15% for noise RMS > 5%. It is also reflected on an increase of the merit function: the RMS error between the traces increases from 0.16 to 6.7% when the noise level varies from 0 to 8%. Also, the spectral phase starts to differ from the simulated phase, especially in the low frequency region where the phase variation is faster, and the SH signal has a lower level. Nevertheless, it is found that even when the minima structures are blurred or have even disappeared, the redundancy of the trace encodes enough information to approximately retrieve the pulse measurement.

### Influence of the phase retardation

The theoretical demonstration of amplitude swing^12^ was done considering an MWP operating as a half-waveplate for the central wavelength (phase retardation $\phi_{0,fs} = \pi$). Since the same plate can be used to measure pulses with different spectra or different plates can be used to measure the same pulse, it is important to study the influence of the phase retardation in the measurement.

For this purpose, we simulated the amplitude swing trace when having $\phi_{0,fs}$ ranging from 0 to $2\pi$. It should be kept in mind that the full relative phase variation is actually ranging from $2\pi m$ to $2\pi (m + 1)$ due to having an MWP. The results are shown in Fig. 3 and Supplementary Video 2. In the SH trace (Fig. 3, Row a) it is shown how the spectral locations of maxima and minima are shifted due to the variation of $\phi_{0,fs}$ (taking into account the particular spectral phase of the pulse).

When retrieved, the convergence is evenly good for the different values of $\phi_{0,fs}$, which is further confirmed by the comparison of the retrieved spectral phases (Fig. 3, Row c) and the retrieved temporal intensities (the statistical error of the pulse duration is kept below 0.2% in all cases) and phases (Fig. 3, Row d) with the simulated inputs. Therefore, we can conclude that the phase retardation of the MWP can be arbitrarily chosen in an experiment. In the experimental results, we will experimentally reach the same conclusion by using 3 different quartz plates.

### Influence of the time delay

With the same motivation than in the previous case, in this section we study the effect of having MWP with different thicknesses, e.g., introducing different delays between the two replicas due to the MWP birefringence. The delay is proportional to the MWP thickness, provided that the plate material is the same. Although here we consider quartz, the choice of material is arbitrary, as long as the thickness can be chosen to reach an appropriate delay. In our previous work^12^, it is theoretically argued that having a delay of the order of the pulse duration is optimum for encoding the pulse phase. Here, we want to explore how tight or relaxed is this condition.

**Figure 7:**
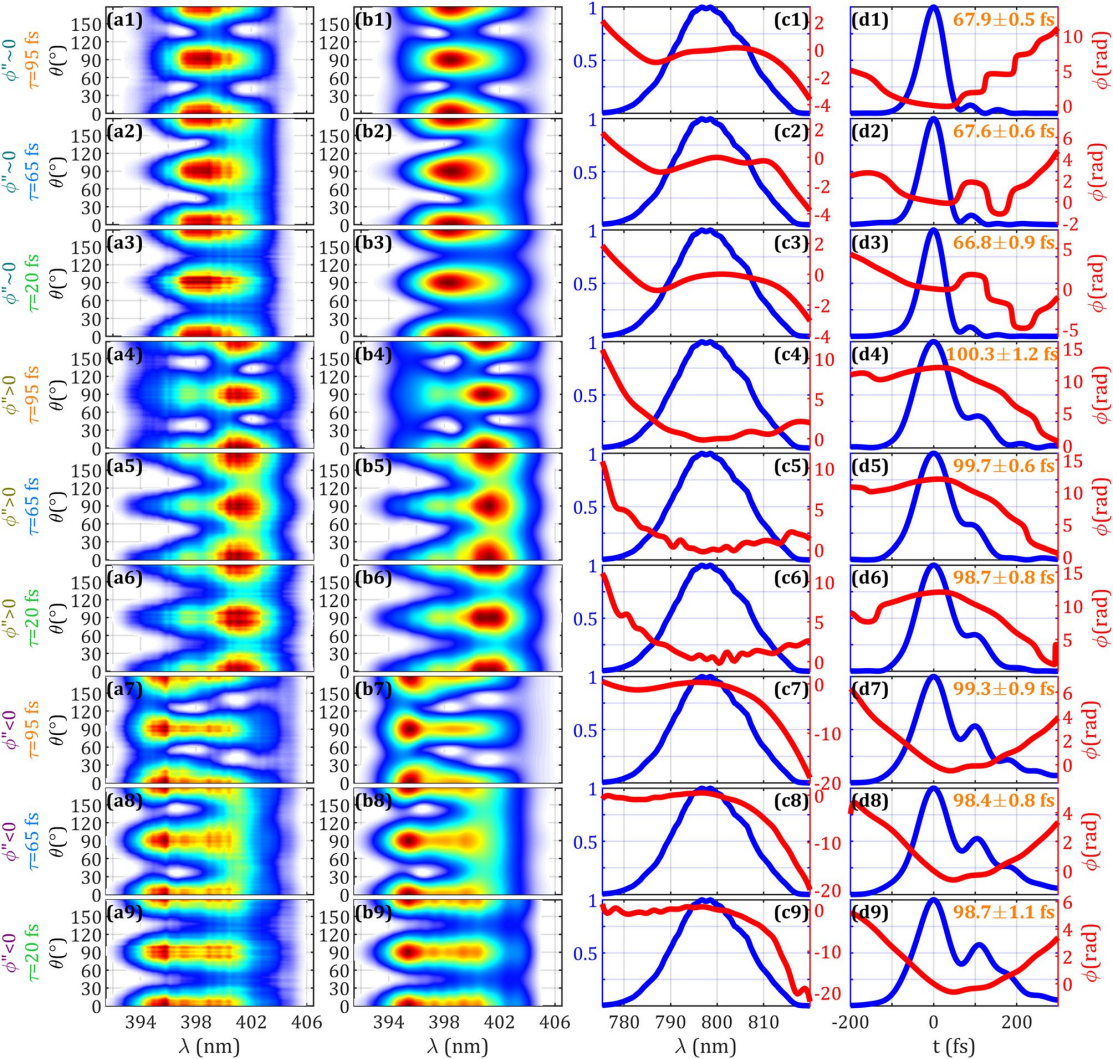
Experimental results of amplitude swing using 3-mm (delay $\tau = 95\,{\text{fs}}$) (rows 1,4,7), 2-mm ($\tau = 65\,{\text{fs}}$) (rows 2,5,8), and 0.65-mm ($\tau = 20\,{\text{fs}}$) (rows 3,6,9) quartz plates. The pulses correspond to around zero linear chirp $\phi ^{{\prime \prime }}$ (rows 1–3) (duration 67.4 fs), positive chirp (rows 4–6) (duration 99.6 fs), and negative chirp (rows 7–9) (duration 98.8 fs). Experimental trace (column **a**), retrieved trace (column **b**), measured spectrum (blue) and retrieved spectral phase (red) (column **c**), retrieved temporal intensity (blue) and phase (red) (column **d**).

In the simulations, we explore different ratios between the pulse Fourier-limit FWHM, $\tau_{\small FL}$, and the delay $\tau$ introduced by the MWP (related to the plate thickness). The results are shown in Fig. 4 and Supplementary Video 3. In the simulated trace it is shown that, for shorter delays, the trace dependence with the spectral phase is smaller, as expected. When increasing the delay, the trace has more spectral structure due to the high-order nature of the MWP. The amplitude swing retrieval works correctly in a wide range of delays, being prudent a delay 3 times larger or smaller than $\tau_{\small FL}$ can be estimated to be valid. In the reconstructions, the pulse retrieval and the convergence are good within this range (RMS error ≤ 0.36%). The statistical error of the retrieved pulse duration is kept well below 1% for all cases with ratio ${\tau \mathord{\left/ {\vphantom {\tau {\tau_{FL} \le 4}}} \right. \kern-\nulldelimiterspace} {\tau_{\small FL} \le 4}}$. The convergence time increases especially for higher values of the delay, although the final convergence and merit function is equally good, which is confirmed by comparison with the simulated input pulse. For the highest value of delay, $\tau = 5\tau_{\small FL}$ (Supplementary Video 3), the retrieval does not complete the convergence (RMS error 4.3%) and the spectral phase for lower frequencies differs from the simulated one. We will show experimental results with different delays in Fig. 7.

### Robustness against spectral clipping

In the present section, we consider the cases of lateral clipping in the lower or higher frequencies of the amplitude swing trace, as well as hollow regions, without signal, in the centre of the trace. This study is interesting as it is possible experimentally having, for example, a limited bandwidth of SH conversion, or part of the SH signal being absorbed by the non-linear material or by the optics or detectors employed. The frequency marginal (SH trace integrated in the angle dimension) expands from 390 to 410 nm, being the maximum at 398 nm and having a FWHM of 9.3 nm.

First, we consider the clipping of low frequencies, in the range between the higher wavelength (410 nm) and the maximum of the SH signal (398 nm). We define the percentage of clipping as the part of this interval in the amplitude swing trace that is artificially set to zero. By means of the simulations, we find that the retrieval is good (convergence time, merit function and retrieved pulse) almost up to the whole clipping, as seen in Fig. 5 (Column 1) and Supplementary Video 4. The retrieved trace is similar to the complete simulated trace (Fig. 5 (b1)), only having small differences in the reddish part of the trace for the final case of 100% low frequency clipping.

Second, we studied the clipping of high frequencies, in the interval between the lower wavelength (390 nm) and the maximum of the SH signal (398 nm), as shown in the results in Fig. 5 (Column 2) and Supplementary Video 5. The retrieval is good up to a clipping of 60% [Fig. 5 (b2)] with pulse duration error < 1%, whereas for higher clippings the retrieved pulses start having small discrepancies in the temporal and spectral phase for the lower intensity part of the pulse intensity and spectrum, respectively. For the highest clippings, the retrieved trace and pulse differ substantially from the simulated ones, while still providing the global structure of the pulse. The impact on the pulse reconstruction of a certain region of spectral clipping may be related to the specific pulse to be measured (spectral amplitude and phase) and the MWP characteristics (mainly the delay and phase retardation), taking into account that those parameters determine the amplitude swing trace structure that encodes more evidently the pulse information.

Then, we simulated the effect of setting to zero the SH trace in a bandwidth centred at 398 nm (indicated by the central frequency $\omega_{c}$) with a total range being a percentage of the SH signal FWHM (i.e., 9.3 nm), which corresponds to the part of the trace having more signal. The results are shown in Fig. 5 (Columns 3 and 4) and Supplementary Video 6 for different cases of hollow regions. We find that the retrieved pulse is good up to 50% of gap (pulse duration error < 1%) and above that value the disagreement increases significantly.

### Calibration and retrieval of the trace using the frequency marginal

In the last set of simulations, we studied the case of having an uncalibrated non-flat spectral response of the SH generation and/or detection, which simultaneously includes the case of having less bandwidth converted than the expected bandwidth (clipping in both spectral sides of the trace).

The aim of these simulations is to demonstrate whether the technique is able to retrieve the pulse measurement in the case of uncalibrated (and clipped) traces. For this purpose, we have calculated the analytical expression of the frequency marginal $M_{\omega } \left( \omega \right)$ of the amplitude swing trace (the integral of the SH trace over the rotation angle). We find that $M_{\omega } \left( \omega \right)$ [see Eq. (8) in the Appendix] depends on the spectral amplitude and phase of the input pulse, and the MWP axes dispersion. As described before, the marginal $M_{\omega }$ can be used to calibrate the SH response while retrieving the pulse characterization.

For the set of simulations, we numerically applied a parabolic spectral response to the simulated trace and then performed the retrieval. This simulated response was centred at 400 nm, having different spectral bandwidths $\Delta \lambda$ (defined as the FWHM of the response function) down to 4 nm, which entails non-flat spectral response and simultaneous spectral clipping in the SH trace (notice that the complete frequency marginal has a FWHM of 9.3 nm).

In Fig. 6 (and Supplementary Video 7), we show the results for different $\Delta \lambda$ in the SH response, including the simulated uncalibrated trace (Row a), the retrieved trace (Row b), the retrieved spectral phase (Row c) and the retrieved temporal intensity and phase (Row d). We found that despite of the spectral clipping and having non-flat spectral response, the pulse was correctly retrieved for SH response bandwidth (FWHM) around half the SH signal bandwidth (FWHM) for perfect phase-matching. Notice that for extreme conditions of clipping the experimental noise could affect the reconstruction. Also, in Fig. 6 (Row e) we show the marginals to make evident the simulated narrower bandwidth (in agreement for the simulated and retrieved traces) compared to the theoretical analytical expression for the marginal with the full bandwidth of the SH signal assuming flat SH response. By comparing these functions, we retrieved the applied SH response function (Fig. 6, Row f), again showing a good agreement with the simulated function. During the tests, we also computed the retrieved marginal from direct integration of the reconstructed trace, then we compared it to the analytical trace, obtaining the same results.

### Experimental results

For the experimental demonstration, we used a chirped pulse amplification (CPA) laser system (Spitfire ACE, Spectra Physics) delivering pulses at 5 kHz repetition rate, centred at 798 nm, with a Fourier-limit duration of $\tau_{\small FL} = 60\,{\text{fs}}$ (FWHM) and 1.6 mJ of energy per pulse. The pulses were linearly polarized in the *x*-axis. As explained before (Fig. 1), the detection system consists of a MWP rotating from 0 to 180º and a linear polarizer selecting the horizontal projection (producing the relative amplitude swing of two delayed replicas), a nonlinear crystal (NL) to produce SH and a linear polarizer to remove the remaining fundamental signal before detection of the SH spectra (fibre spectrometer HR4000, Ocean Optics Inc.). The spectrum of the fundamental pulse was measured by a fibre spectrometer (AvaSpec 2048-USB1, Avantes Inc.). We did not focus the beam onto the NL crystal because the intensity was high enough to efficiently produce SH. In the present experiments, the SH produced was type I, so we used a linear polarizer to filter out the fundamental signal. The dispersion introduced by the MWP^20^ and the first linear polarizer (before the NL crystal) was corrected within the retrieval algorithm.

To experimentally study the influence of the phase retardation and the replica delay in the pulse measurement and retrieval, we used three quartz MWPs with different thickness. We chose thicknesses introducing arbitrary phase retardations $\phi_{0,fs}$ (defined at 800 nm), and different delays $\tau$ similar to, below and above the $\tau_{\small FL} = 60\;{\text{fs}}$ duration of the pulse. The first MWP with 3-mm thickness introduces a delay $\tau = 95\,{\text{fs}}$ and phase retardation $\phi_{0,fs} = 0.97\pi$, therefore having near half-wave plate operation at the central wavelength. The second MWP had a 2-mm thickness, introducing $\tau = 65\,{\text{fs}}$ and $\phi_{0,fs} = 0.34\pi$. The third MWP had a 0.65-mm thickness, introducing $\tau = 20\,{\text{fs}}$ and $\phi_{0,fs} = 0.50\pi$ (quarter-wave plate operation). With those MWPs, we compared the measurements of three different pulses, around optimum compression (slight remaining third order dispersion), and with two values respectively of positive and negative chirp ($\phi ^{{\prime \prime }}$) introduced by the CPA grating compressor. The NL crystal employed was a type I BBO crystal with full-bandwidth phase-matching due to the 20-μm thickness.

The results of the measured and retrieved data are shown in Fig. 7 in different rows for the three different pulses and the three different MWPs. The retrieved SH traces (column b) converge correctly to the experimental SH traces (column a). Each measurement is retrieved 5 times: in Fig. 7 (column d) it is given the average value and the associated error (standard deviation) for the retrieved pulse duration (FWHM). For each measurement, the retrieved pulse durations are consistent with respect to the calculated error intervals for the same of the 3 pulses measured with the 3 different plates. From those results, we calculate the average of the retrieved pulse durations. For the pulse with the lowest $\phi ^{{\prime \prime }}$ is 67.4 ± 0.7 fs, while for the positive chirp case is 99.6 ± 0.9 fs and for the negative chirp is 98.8 ± 0.9 fs. It is found that the spectral phase of the pulse is encoded in the amplitude swing trace for the three MWPs employed independently on the delay, particularly showing the suitability of using delays from $\tau = 0.3\,\tau_{\small FL}$ to $\tau = 1.5\,\tau_{\small FL}$. It is also experimentally shown that different phase retardations can be employed, e.g. standard half or quarter MWPs, but also other arbitrary intermediate retardations.

**Figure 8:**
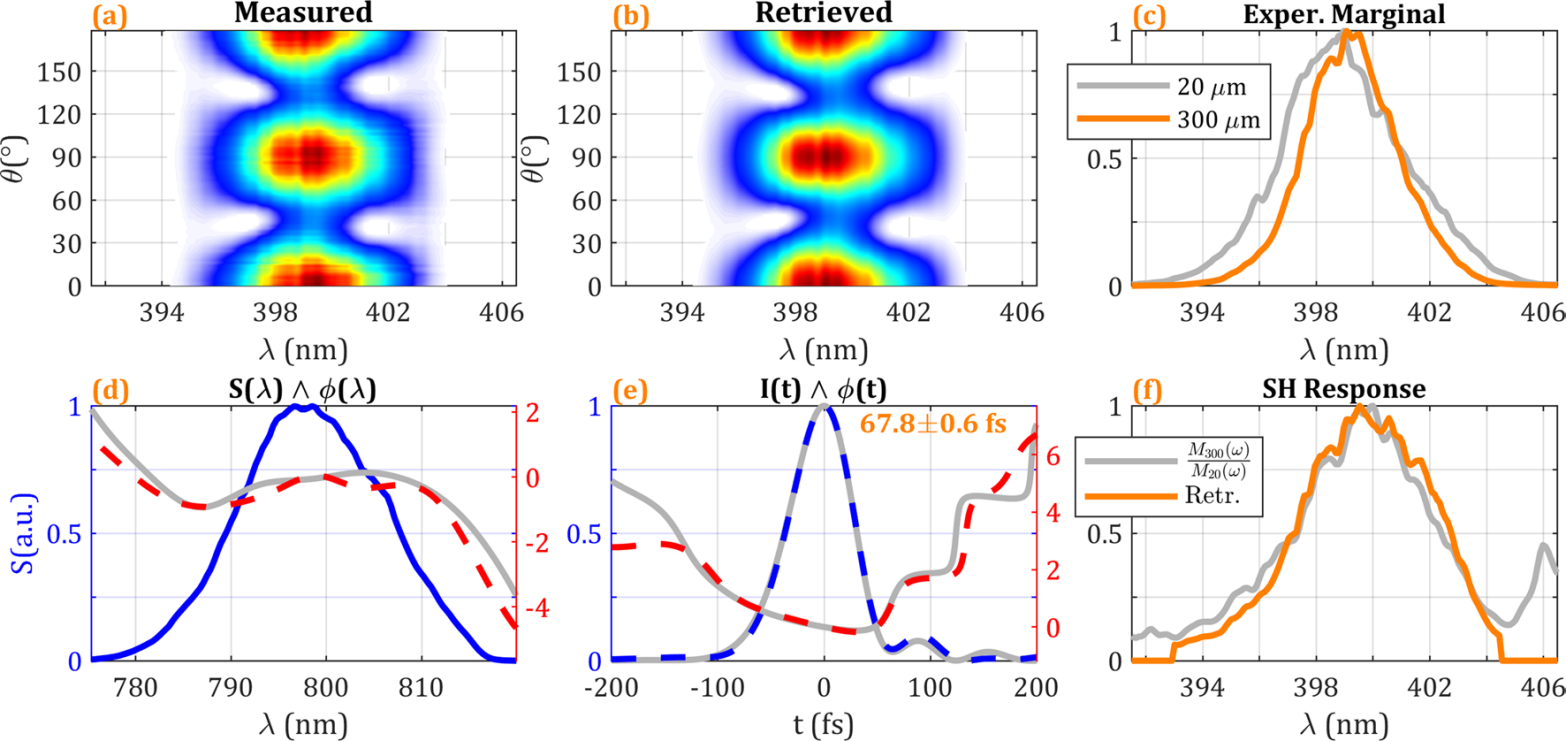
Measurement of the pulse for optimum compression (corresponding to Fig. 7, row 1) with a thicker BBO crystal of 300 μm. (**a**) Experimental and (**b**) retrieved amplitude swing traces. (**d**) Spectrum and retrieved phase (dashed red) compared to 20-μm BBO retrieval (grey). (**e**) Retrieved temporal intensity (dashed blue) and phase (dashed red) compared to 20-μm BBO retrieval (grey). (**c**) Comparison of experimental frequency marginals for 300-μm (orange) and 20-μm (grey) BBO crystals. (**f**) SH response of the 300-μm BBO calculated within the optimization retrieval (orange) compared to the calculated SH response from the marginals in (**c**) (grey).

Finally, we experimentally tested, with the previous pulse having around zero linear chirp (around 67-fs pulses) and using the previous 3-mm quartz MWP (95-fs delay), the effect of having limited phase-matching bandwidth by using a 300-μm thickness BBO crystal (compared to the previous 20-μm BBO) to generate the SH signal (same conditions than Fig. 7, row 1, except for the BBO thickness). Consequently, the nonlinear signal is narrower, which can be seen in the experimental trace (Fig. 8a) and in the comparison of the experimental frequency marginals for both BBO crystals (Fig. 8c). We did the retrieval of the SH as described in Eq. (2) and in the previous section, using the theoretical expression [analytical Eq. (8)] for the frequency marginal to calibrate the SH response. The calibration retrieved by the optimization algorithm is shown in Fig. 8f, which agrees with the predicted response calculated from the ratio between the experimental marginals shown in Fig. 8c. The retrieved pulse in the spectral domain (Fig. 8d) and the temporal domain (Fig. 8e) is in good agreement with the results obtained for a 20-μm BBO with flat spectral response using the same MWP (corresponding to Fig. 7, row 1), the retrieved duration (67.8 ± 0.6 fs) falls within the previously found interval 67.9 ± 0.5 fs.

### Few-cycle pulse regime

**Figure 9:**
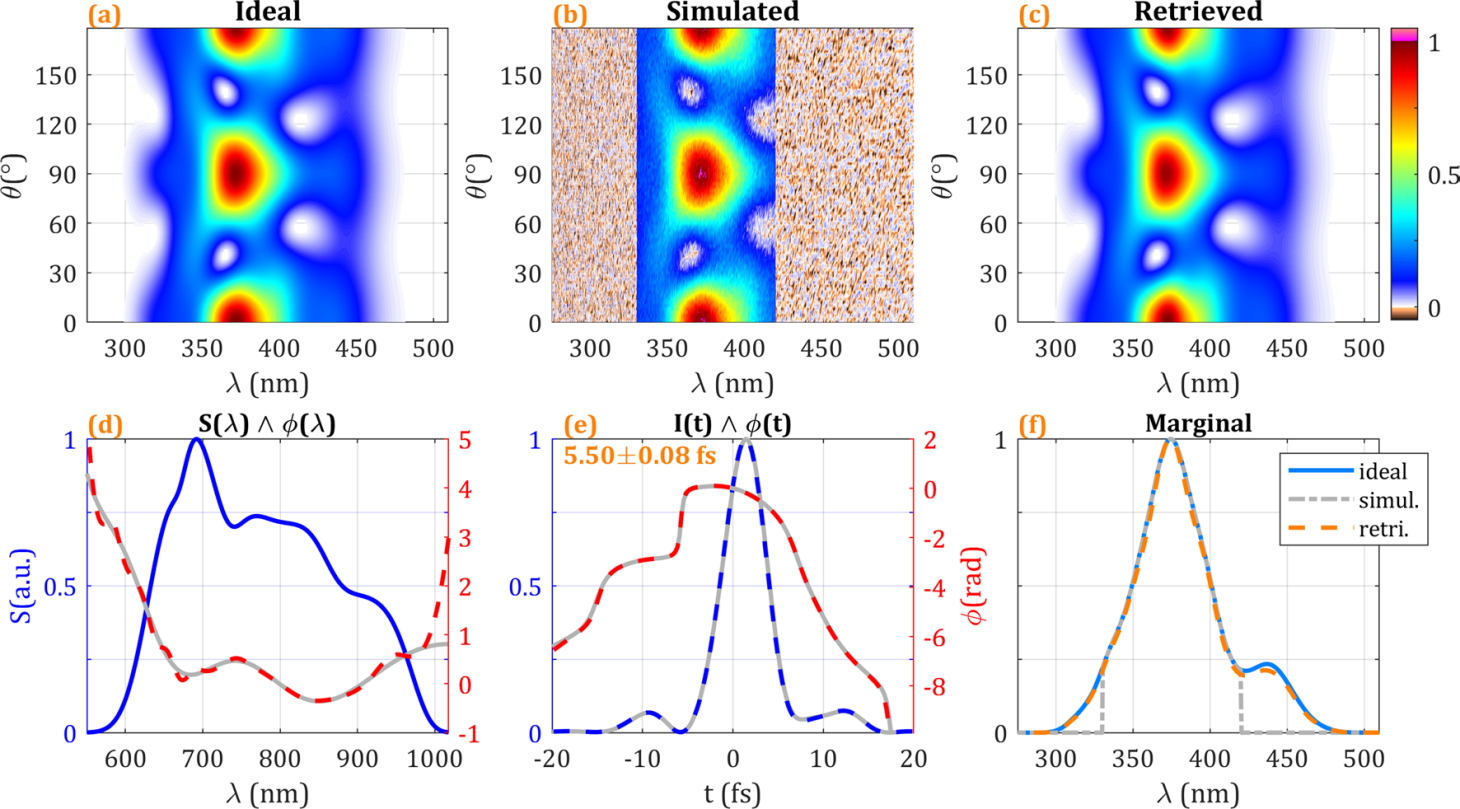
(**a**) Ideal, (**b**) added-noise and spectrally clipped, and (**c**) retrieved amplitude swing traces for a few-cycle pulse. (**d**) Simulated spectral amplitude (blue) and simulated (grey) and retrieved (dashed red) spectral phase. (**e**) Retrieved temporal intensity (dashed blue) and phase (dashed red) compared to the simulated pulse (grey). (**f**) Frequency marginals of the ideal (blue), noisy and clipped simulated (dashed grey) and retrieved (dashed orange) traces.

To obtain a more complete insight of the potential of the amplitude swing approach, we have finally explored its application and robustness in the few-cycle pulse regime (Fig. 9). The pulse to be retrieved presents a Fourier Transform limit duration of 4.65 fs (FWHM) and a quite flat spectral phase, yielding 5.54 fs (FWHM) pulse duration (the phase is simulated with a combination of second- and third-order dispersion of 5 fs^2^ and 9 fs^3^, and an oscillatory sinusoidal phase with 0.4 rad amplitude). In order to check the method robustness, it has been added a 2.4% RMS noise to the amplitude swing trace and the SH spectra have been clipped below 330 nm and above 420 nm, featuring the problems arising from the analysis of such broadband pulses and the nonlinear material phase-matching.

Concerning the amplitude swing setup, the considered MWP is a 300 μm-thick quartz plate, introducing a delay between the extraordinary and ordinary components of 9.44 fs, around twice the Fourier limit duration (please note that, while being an acceptable MWP delay, it is not the optimal one, which would be around the Fourier Transform limit duration^12^). This amount of material introduces dispersion, being relevant in this few-cycle regime: the pulse varies from 5.54 fs before the MWP to 9.2 fs (fast axis) and 9.4 fs (slow axis) after the MWP. However, using the dispersion of both axes (known by the material Sellmeier expression or by direct calibration using spectral interferometry^22^), this dispersive effect can be correctly taken into account in the retrieval algorithm. The phase retardation of the MWP is set to an intermediate value of $\phi_{0,fs} = 0.8\pi$ at 800 nm.

Figure 9a shows the ideal amplitude swing trace (without noise nor clipping) corresponding to the considered few-cycle pulse. In Fig. 9b, the noise and spectral clipping have been introduced, distorting the amplitude swing trace. However, when the retrieval algorithm is applied to the latter, the ideal amplitude swing trace is completely recovered, as shown in Fig. 9c. Indeed, Fig. 9d shows the retrieved spectral phase (dashed red) matching very well to the input pulse (grey). Consequently, in Fig. 9e the retrieved temporal intensity (dashed blue) and phase (dashed red) match to the simulated input pulse (grey). It has been considered 10 different retrievals with random guess phases, in order to give an idea of the retrieval error. The retrieved pulse shows a duration of 5.50 ± 0.08 fs (FWHM), in good agreement with the original 5.54 fs duration of the simulated input pulse. When analysing the amplitude swing trace marginal (Fig. 9f), the corresponding to the retrieved trace (dashed orange) fits well with the one corresponding to the ideal trace from the simulated input pulse (blue), even if the marginal from the noisy and clipped trace used to apply the algorithm is considerably clipped (dashed grey).

Therefore, the amplitude swing procedure is also well suit for retrieving few-cycle regime pulses even when the traces exhibit spectral clipping and some moderate noise. Finally, the future study of the validity of the technique at other spectral ranges is also interesting. In the Supplementary Sect. S2, we show an example of the simulation of retrieved pulses in the mid-infrared.

## Conclusions

We have studied the robustness of amplitude swing measurements, finding tolerance to the presence of noise above 5% (noise to signal), benefited from redundancy in the data. Then, we have explored the influence of the two key parameters in the detection. We found that the phase retardation does not have a relevant role, while the delay between the replicas can be used in a flexible range below and above the Fourier-limit duration of the pulse to be measured. These properties ease the suitability of a particular multi-order waveplate to be used with pulses of different temporal and spectral ranges. For this purpose, we performed numerical simulations and experimental measurements with different pulses and with different plates.

Also, thanks to the redundancy in the two-dimensional trace, we demonstrated that the retrieval algorithm can operate with incomplete traces, presenting spectral clipping or hollow regions, which is an important property in certain applications.

We demonstrated that it is possible to use the frequency marginal depending on the spectral phase of the pulse to calibrate in amplitude the experimental trace, i.e. the second-harmonic response. We derived the analytical expression for that marginal and used it to retrieve that response within the iterative retrieval algorithm, updating in every step the marginal and the trace calibration. We showed it theoretically for responses with different bandwidths and experimentally by using a thicker nonlinear crystal with non-flat response. In the case of a calibrated trace, the marginal could be used to assess the quality of the measured or the retrieved trace.

In addition, the technique has been theoretically tested in the few-cycle pulse regime considering noise and spectral clipping issues. The above commented properties of the amplitude swing approach allow a good retrieval of the pulses even at that demanding pulse duration regime.

The conclusions extracted from this study must be understood as a qualitative route of what is expected for a general case because, depending on the particular pulse to be measured, it may be more or less difficult to retrieve the pulse information in an adverse scenario.

### Electronic supplementary material

Below is the link to the electronic supplementary material.

Supplementary Information 1.

Supplementary Video 1.

Supplementary Video 2.

Supplementary Video 3.

Supplementary Video 4.

Supplementary Video 5.

Supplementary Video 6.

Supplementary Video 7.

## Data Availability

The datasets generated and/or analysed during the current study are available from the corresponding author on reasonable request.

## Appendix: analytical expression for the frequency marginal

In this Appendix, we demonstrate the analytical expression for the frequency marginal. For this purpose, we consider the case of a linearly polarized (*x*-axis) input pulse $E_{0} \left( \omega \right) = A\left( \omega \right)e^{{i\phi_{p} (\omega )}}$, with spectral amplitude $A\left( \omega \right)$ and phase $\phi_{p} (\omega )$. Then, we apply the MWP with its fast axis oriented at an angle $\theta$ with respect to the *x*-axis, where we call $\phi_{f} (\omega )$ and $\phi_{s} (\omega )$, the spectral phase (dispersion) introduced by the fast and slow axes, respectively. Then, a horizontal linear polarizer selects the *x*-axis projection of the beam, which can be obtained with Jones calculus

3 $$E_{\it x} \left( {\omega ,\theta } \right) = \left[ {e^{{i\phi_{f} (\omega )}} \cos^{2} \theta + e^{{i\phi_{s} (\omega )}} sin^{2} \theta } \right]E_{0} \left( \omega \right) = E_{0f} \left( \omega \right)\cos^{2} \theta + E_{0s} \left( \omega \right)sin^{2} \theta$$

where $E_{0f} \left( \omega \right) = A\left( \omega \right)e^{{i\phi_{p} (\omega )}} e^{{i\phi_{f} (\omega )}}$ and $E_{0s} \left( \omega \right) = A\left( \omega \right)e^{{i\phi_{p} (\omega )}} e^{{i\phi_{s} (\omega )}}$ represent the input pulse after passing through the fast and slow axes of the MWP, respectively.

Then, the pulse given by Eq. (3) generates second harmonic (SH), which we compute analytically as

4 $$E_{SH} \left( {2\omega ,\theta } \right) = \int\limits_{ - \infty }^{\infty } {E_{\it x} \left( {\omega - \Omega ,\theta } \right)E_{\it x} \left( {\omega + \Omega ,\theta } \right)d\Omega }$$

The spectrum of the SH signal $S_{SH} \left( {2\omega ,\theta } \right)$ is calculated from Eqs. (3,4) integrating with respect to $\Omega_{1}$ and $\Omega_{2}$

5 $$\begin{gathered} S_{SH} \left( {2\omega ,\theta } \right) = E_{SH} \left( {2\omega ,\theta } \right)E_{SH}^{*} \left( {2\omega ,\theta } \right) \hfill \\ \,\,\,\,\,\,\, = \int\limits_{{\Omega_{1} = - \infty }}^{\infty } {\int\limits_{{\Omega_{2} = - \infty }}^{\infty } {d\Omega_{1} d\Omega_{2} } } E_{\it x} \left( {\omega - \Omega_{1} ,\theta } \right)E_{\it x} \left( {\omega + \Omega_{1} ,\theta } \right)E_{\it x}^{*} \left( {\omega - \Omega_{2} ,\theta } \right)E_{\it x}^{*} \left( {\omega + \Omega_{2} ,\theta } \right) \hfill \\ \,\,\,\,\,\,\, = \left| {E_{ff} \cos^{4} \theta + 2E_{fs} \cos^{2} \theta \sin^{2} \theta + E_{ss} \sin^{4} \theta } \right|^{2} \hfill \\ \end{gathered}$$

where we denote by $E_{ff}$, $E_{ss}$ and $E_{fs}$ the electric field (in the frequency domain) of the SH of the pulse $E_{0f} \left( \omega \right)$, the pulse $E_{0s} \left( \omega \right)$, and their sum of frequency, respectively

6 $$\begin{aligned} E_{{ff}} \left( {2\omega } \right) & = \int\limits_{{ - \infty }}^{\infty } {E_{{0f}} \left( {\omega - \Omega } \right)E_{{0f}} \left( {\omega + \Omega } \right)d\Omega } \\ E_{{ss}} \left( {2\omega } \right) & = \int\limits_{{ - \infty }}^{\infty } {E_{{0s}} \left( {\omega - \Omega } \right)E_{{0s}} \left( {\omega + \Omega } \right)d\Omega } \\ E_{{fs}} \left( {2\omega } \right) & = \int\limits_{{ - \infty }}^{\infty } {E_{{0f}} \left( {\omega - \Omega } \right)E_{{0s}} \left( {\omega + \Omega } \right)d\Omega } \\ \end{aligned}$$

then, the SH signal can be expanded and rewritten to

7 $$\begin{aligned} S_{{SH}} \left( {\omega ,\theta } \right) & = \left| {E_{{ff}} } \right|^{2} \cos ^{8} \theta + \left| {E_{{ss}} } \right|^{2} \sin^{8} \theta + 4\left| {E_{{fs}} } \right|^{2} \cos ^{4} \theta \sin ^{4} \theta + \left( {E_{{ff}} E_{{ss}}^{*} + E_{{ff}}^{*} E_{{ss}} } \right)\cos ^{4} \theta \sin ^{4} \theta \\ & \quad + 2\left( {E_{{ff}} E_{{fs}}^{*} + E_{{ff}}^{*} E_{{fs}} } \right)\cos ^{6} \theta \sin ^{2} \theta + 2\left( {E_{{ss}} E_{{fs}}^{*} + E_{{ss}}^{*} E_{{fs}} } \right)\cos ^{2} \theta \sin ^{6} \theta \\ \end{aligned}$$

The frequency marginal $M_{\omega }$ is defined as the integral of the amplitude swing trace, Eq. (7), with respect to the rotation angle $\theta$. Integrating, grouping terms and simplifying, gives the final expression for the frequency marginal

8 $$M_{\omega } \left( \omega \right) = \int\limits_{0}^{\pi } {S_{SH} \left( {2\omega ,\theta } \right)d\theta } = \frac{\pi }{128}\left\{ {35S_{ff} + 35S_{ss} + 12S_{fs} + 6\Re \left[ {E_{ff} E_{ss}^{*} } \right] + 20\Re \left[ {\left( {E_{ff} + E_{ss} } \right)E_{fs}^{*} } \right]} \right\}$$

in which some terms can be directly interpreted, while others are introduced here for the only purpose of calculating the frequency marginal of the pulse to be measured. The terms $S_{ff} = \left| {E_{ff} } \right|^{2}$ and $S_{ss} = \left| {E_{ss} } \right|^{2}$ represent, respectively, the spectrum of the SH of the pulses $E_{0f} \left( \omega \right)$ and $E_{0s} \left( \omega \right)$, while $S_{fs} = \left| {E_{fs} } \right|^{2}$ is the spectrum of the sum of frequency (can be interpreted as SH) of both pulses, $E_{0f} \left( \omega \right)$ and $E_{0s} \left( \omega \right)$. The term $\Re \left[ {E_{ff} E_{ss}^{*} } \right]$ is the real part of the SH of $E_{0f} \left( \omega \right)$ and the conjugated SH of $E_{0s} \left( \omega \right)$, and involves the relative dispersion of the slow and fast axes of the MWP. Finally, the term $\Re \left[ {\left( {E_{ff} + E_{ss} } \right)E_{fs}^{*} } \right]$ involves the electric field of the SH of $E_{0f} \left( \omega \right)$ and $E_{0s} \left( \omega \right)$, and the conjugated sum of frequency of $E_{0f} \left( \omega \right)$ and $E_{0s} \left( \omega \right)$.
