# Supplementary material for: Robustness and capabilities of ultrashort laser pulses characterization with amplitude swing
Source: Sci Rep. 2020 Oct 27;10:18364. doi: 10.1038/s41598-020-75220-4 (PMC7591537; doi:10.1038/s41598-020-75220-4)
Supplement: Supplementary file 1 — Supplementary Information 1. [file 41598_2020_75220_MOESM1_ESM.pdf]

Supplementary Information

Robustness and capabilities of ultrashort laser pulses characterization with amplitude swing

Íñigo J. Sola and Benjamín Alonso*

Grupo de Investigación en Aplicaciones del Láser y Fotónica, Departamento de Física Aplicada, University of Salamanca, E-37008 Salamanca, Spain
*Corresponding author: b.alonso@usal.es

S1. Description of the Supplementary Videos.
The manuscript is accompanied by the Supplementary Videos described below.

Supplementary Video 1

Effect on the SH trace of different noise levels is represented in different snapshots (associated with Figure 2 in the manuscript). (a) Simulated and (b) retrieved amplitude swing traces. (c) Spectrum (blue), simulated (grey) and retrieved (dashed red) spectral phases. (d) Simulated temporal intensity and phase (grey), retrieved temporal intensity (dashed blue) and phase (dashed red). Inset: retrieved intensity FWHM.

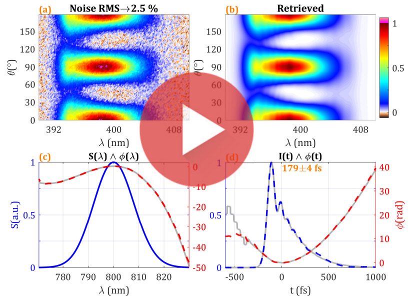

Supplementary Video 2

Effect on the SH trace of different phase retardations for the central wavelength of the MWP is represented in different snapshots (associated with Figure 3 in the manuscript). (a) Simulated and (b) retrieved amplitude swing traces. (c) Spectrum (blue), simulated (grey) and retrieved (dashed red) spectral phases. (d) Simulated temporal intensity and phase (grey), retrieved temporal intensity (dashed blue) and phase (dashed red). Inset: retrieved intensity FWHM.

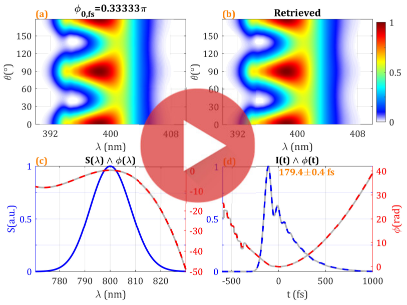

Supplementary Video 3

Effect on the SH trace of different temporal delays (due to different plate thicknesses) introduced by the MWP is represented in different snapshots (associated with Figure 4 in the manuscript). (a) Simulated and (b) retrieved amplitude swing traces. (c) Spectrum (blue), simulated (grey) and retrieved (dashed red) spectral phases. (d) Simulated temporal intensity and phase (grey), retrieved temporal intensity (dashed blue) and phase (dashed red). Inset: retrieved intensity FWHM.

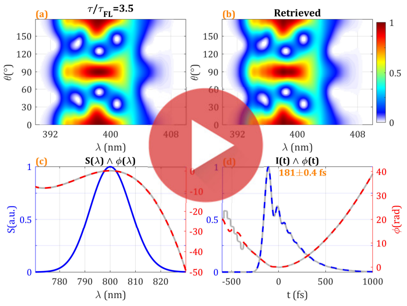

Supplementary Video 4

Effect on the SH trace of lower frequencies spectral clipping is represented in different snapshots (associated with Figure 5, Column 1, in the manuscript). (a) Simulated and (b) retrieved amplitude swing traces. (c) Spectrum (blue), simulated (grey) and retrieved (dashed red) spectral phases. (d) Simulated temporal intensity and phase (grey), retrieved temporal intensity (dashed blue) and phase (dashed red). Inset: retrieved intensity FWHM.

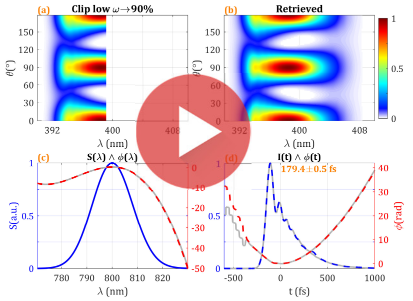

Supplementary Video 5

Effect on the SH trace of higher frequencies spectral clipping is represented in different snapshots (associated with Figure 5, Column 2, in the manuscript). (a) Simulated and (b) retrieved amplitude swing traces. (c) Spectrum (blue), simulated (grey) and retrieved (dashed red) spectral phases. (d) Simulated temporal intensity and phase (grey), retrieved temporal intensity (dashed blue) and phase (dashed red). Inset: retrieved intensity FWHM.

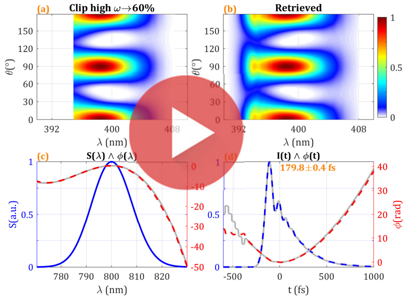

Supplementary Video 6

Effect on the SH trace of hollow central regions spectral clipping is represented in different snapshots (associated with Figure 5, Columns 3-4, in the manuscript). (a) Simulated and (b) retrieved amplitude swing traces. (c) Spectrum (blue), simulated (grey) and retrieved (dashed red) spectral phases. (d) Simulated temporal intensity and phase (grey), retrieved temporal intensity (dashed blue) and phase (dashed red). Inset: retrieved intensity FWHM.

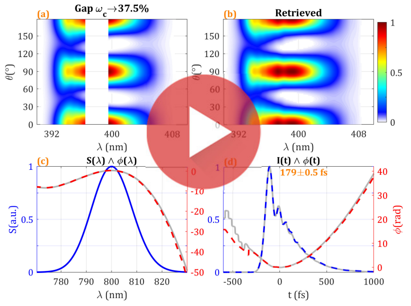

Supplementary Video 7

Effect on the SH trace of uncalibrated amplitude due to non-flat SH response is represented in different snapshots (associated with Figure 6 in the manuscript). (a) Simulated and (b) retrieved amplitude swing traces. (d) Spectrum (blue), simulated (grey) and retrieved (dashed red) spectral phases. (e) Simulated temporal intensity and phase (grey), retrieved temporal intensity (dashed blue) and phase (dashed red). (c) Marginal of the simulated measured trace shown in (a) (grey) and marginal of the retrieved trace shown in (b) (dashed blue) integrating over the rotation angle, compared to the retrieved marginal using the analytical expression and the retrieved spectral phase (dashed orange). (f) SH response applied to the simulated trace (grey) and retrieved SH response using the marginals (dashed orange). Inset: retrieved intensity FWHM.

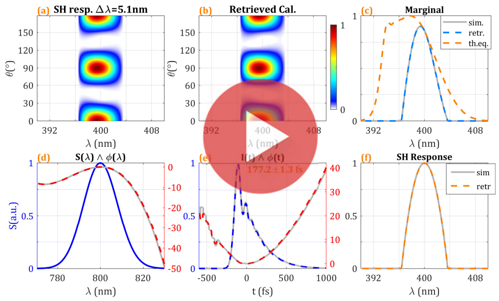

S2. Application to mid-infrared pulses.

The application of the technique in other spectral ranges is a very interesting point. We have explored the performance of the technique with an example in the region of interest for telecommunications. We have considered a pulse exhibiting a Gaussian shape spectrum centred at 1500 nm, with 40 nm (full width at half maximum, FWHM) bandwidth, so that its Fourier Transform limit is 59.3 fs (FWHM). The multiple-order waveplate used in the simulations was a 2.4-mm quartz plate that introduced a delay of 74.8 fs between the pulse replicas and operated as half-waveplate for the central wavelength of the spectrum. Concerning the spectral phase, we have considered second- and third-order dispersion of –500 fs^2 and 1000 fs^3, respectively, as well as an oscillatory component with 0.3 rad amplitude. The simulated pulse had a duration of 63.2 fs (FWHM) and the retrieved pulse 63.1 fs (FWHM), as shown below in the Supplementary Figure S1, so that we found that the amplitude swing scheme also works in this spectral domain.

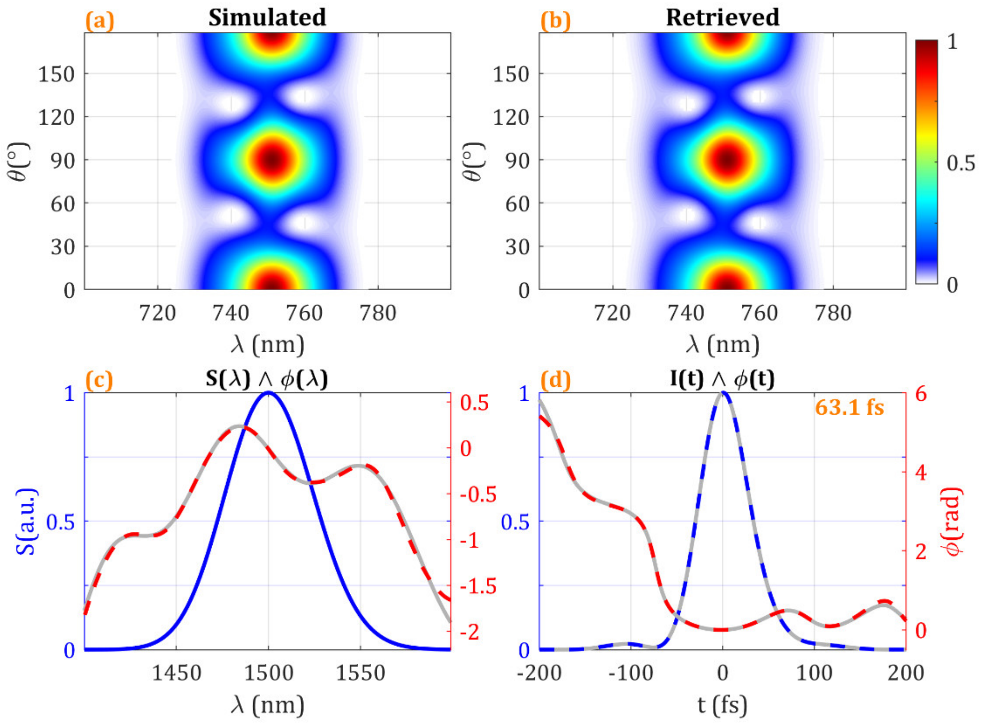

Supplementary Figure S1. Simulation of a mid-infrared pulse. (a) Simulated and (b) retrieved amplitude swing traces. (c) Spectrum (blue), simulated (grey) and retrieved (dashed red) spectral phases. (d) Simulated temporal intensity and phase (grey), retrieved temporal intensity (dashed blue) and phase (dashed red).
